# Acupuncture for primary autoimmune cerebellar ataxia: A CARE-compliant case report with two-year follow-up

**DOI:** 10.1097/MD.0000000000045895

**Published:** 2025-11-14

**Authors:** Zhijie Xu, Fan Xu, Guiping Li, Linna Wu, Haolong Guo, Shu Wang

**Affiliations:** a First Teaching Hospital of Tianjin University of Traditional Chinese Medicine, Tianjin, China; b National Clinical Research Center for Chinese Medicine Acupuncture and Moxibustion, Tianjin, China; c Tianjin Academy of Traditional Chinese Medicine Affiliated Hospital, Tianjin, China.

**Keywords:** acupuncture, case report, primary autoimmune cerebellar ataxia (PACA), supplementary therapy

## Abstract

**AbstractRationale:**

Primary autoimmune cerebellar ataxia (PACA) is an immune-mediated ataxia often lacking disease-specific antibodies, making timely therapy challenging. We explored whether integrative acupuncture might aid recovery alongside immunomodulation.

**Patient concerns:**

A 28-year-old woman presented with right lower limb weakness, gait stiffness and unsteadiness, dysarthria, and intention tremor.

**Diagnoses:**

The patient was diagnosed with PACA and had a history of immunosuppressive therapy.

**Interventions:**

In addition to ongoing immunomodulatory treatment, the patient received a standardized acupuncture regimen delivered during hospitalization and continued as maintenance in the outpatient setting.

**Outcomes:**

Speech fluency and motor coordination improved markedly. At discharge, the Scale for the Assessment and Rating of Ataxia decreased from 20 to 14, and the modified Rankin Scale improved from 3 to 2. Clinical symptoms remained stable at the 2-year follow-up, without reported adverse events.

**Lessons:**

Integrative acupuncture may be a feasible adjunct in PACA, particularly for enhancing motor coordination and functional status; controlled studies are warranted to confirm efficacy and define indications.

## 1. Introduction

Primary autoimmune cerebellar ataxia (PACA) is a subtype of immune-mediated cerebellar ataxia, typically characterized by gait and limb ataxia, dysarthria, and occasionally extrapyramidal symptoms.^[1]^ Diagnosis is often challenging due to the absence of disease-specific autoantibodies, potentially leading to delayed initiation of appropriate therapy.^[2]^ Although PACA has been widely reported, there is a lack of detailed, long-term data on complementary therapies, particularly acupuncture, in patients with progressive cerebellar atrophy despite immunosuppression. Herein, we report a case of PACA treated with acupuncture, in which the patient demonstrated marked symptomatic improvement over a 2-year follow-up period, addressing a critical gap in the literature regarding adjunctive management strategies.

## 2. Case presentation

### 2.1. Clinical presentation

A 28-year-old woman was admitted in February 2023 with a 17-month history of progressive gait instability and dysarthria. Her symptoms initially began in September 2021 with the sudden onset of right lower limb weakness, gait stiffness, and unsteadiness. These symptoms gradually progressed to involve the upper limbs and were accompanied by intention tremor and scanning speech. Initial brain MRI revealed bilateral periventricular hyperintensities, while cerebrospinal fluid analysis showed mild pleocytosis and positive oligoclonal bands. Transient positivity for herpes simplex virus type 1 and cytomegalovirus were detected; however, she exhibited no clinical signs of encephalitis. Further evaluations, including viral panels, paraneoplastic antibodies, autoimmune encephalitis antibodies, EEG, and EMG, were unremarkable.

She was initially diagnosed with general cerebellar ataxia and received intravenous immunoglobulin which provided no clinical benefit. A subsequent course of high-dose methylprednisolone pulse therapy, tapered to oral prednisone, combined with acyclovir, resulted in partial symptom relief. Follow-up MRIs revealed bilateral pyramidal tract and centrum semiovale lesions, and progressive cerebellar atrophy. Autoimmune panel, metabolic screening, and genetic testing were unremarkable.

She was started on oral mycophenolate mofetil 0.5 g BID, later titrated to 0.75 g/0.5 g daily in combination with 10 mg prednisone, which stabilized her condition but did not restore independent ambulation. At the time she sought integrative treatment including acupuncture, her neurological examination revealed persistent ataxia. The Scale for the Assessment and Rating of Ataxia (SARA; total score 20) showed a total patient score of 20, with item scores of gaits 6/8, stance 4/6, sitting 2/4, speech disturbance 3/6, finger chase 1/4, nose-finger test 1/4, fast alternating hand movements 1/4, and heel-shin slide 2/4, reflecting moderate-to-severe gait instability, impaired balance, and dysarthria.

### 2.2. Diagnosis

Given absence of disease-specific autoantibodies, and imaging/cerebrospinal fluid findings, a diagnosis of PACA was established according to the 2020 expert consensus, which is a key diagnostic challenge in this field.^[2]^

### 2.3. Treatment

**Figure 1.**
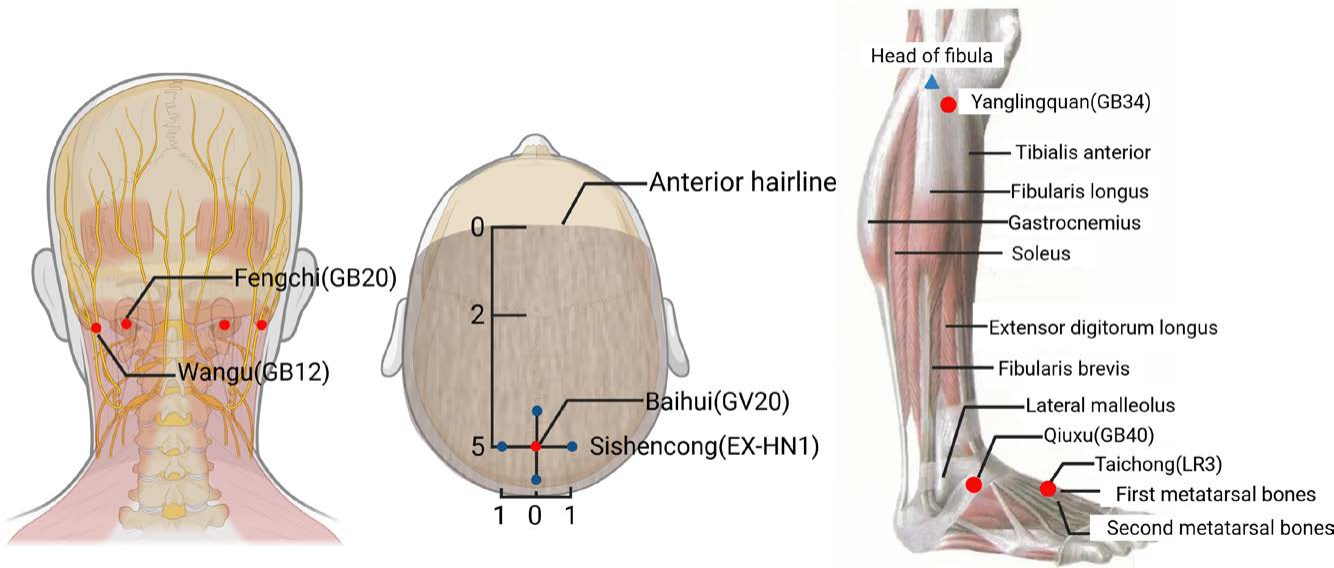
Location of acupoints used in acupuncture treatment. Created using BioRender.

In addition to immunosuppressive therapy, the patient received acupuncture targeting the following main acupoints: Wangu (GB12), Fengchi (GB20), Yanglingquan (GB34), Taichong (LR3), and Qiuxu (GB40), with supporting points Baihui (GV20), Sishencong (EX-HN1; Fig. 1). The patient received acupuncture in a seated position. Sterile disposable needles (φ0.25 × 25 mm) were inserted bilaterally at selected acupoints with standard manual stimulation to elicit the de qi sensation. Each session lasted 20 minutes, and was administered 6 times per week for a total of 3 weeks. This was followed by maintenance acupuncture therapy, delivered 3 times per week in an outpatient setting, and continued for approximately 1 year.

### 2.4. Clinical outcome

**Figure 2.**
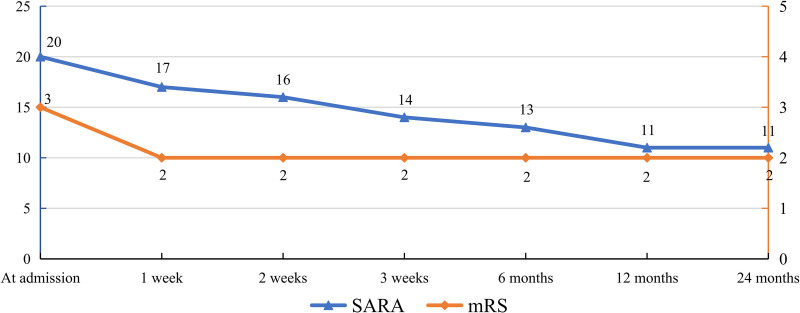
Functional outcome evaluation using the Scale for the Assessment and Rating of Ataxia (SARA) and the modified Rankin Scale (mRS) at 2-year follow-up.

**Figure 3.**
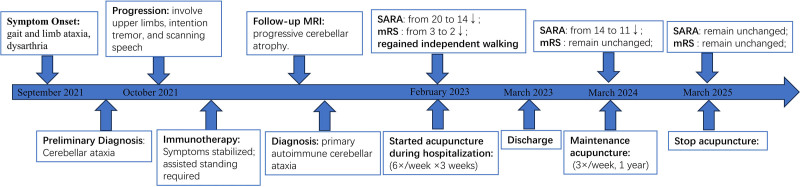
Timeline of clinical course and interventions in a patient with primary autoimmune cerebellar ataxia. mRS = modified Rankin Scale, SARA = Scale for the Assessment and Rating of Ataxia.

The SARA and the modified Rankin Scale (mRS) are among the most commonly clinical tools for evaluating the severity and functional impact of ataxia. After 3 weeks of acupuncture therapy, the patient showed significant improvement in both speech fluency and motor coordination. She regained the ability to walk independently without assistance. A reduction of 1.5 points in the SARA score and 1 point in the mRS score is generally regarded as clinically meaningful. At discharge, the patient exhibited a notable decrease in the SARA score from 20 to 14 and an improvement in the mRS score from 3 to 2, indicating a favorable response to acupuncture (Fig. 2).^[3]^ The observed improvements in the SARA sub-scores – gait from 6 to 4, stance from 4 to 3, and sitting from 2 to 1 – reflect enhanced limb coordination, which corresponded with the patient regaining the ability to walk independently. After 1 year of acupuncture treatment, the patient’s SARA score improved to 11. At the 2-year follow-up, the symptoms remained stable without aggravation. No adverse events occurred throughout the course of acupuncture treatment. The patient’s clinical course, diagnostic evaluations, and sequential therapeutic interventions are summarized in a timeline (Fig. 3).

## 3. Discussion

PACA involves progressive and irreversible damage to Purkinje cells. According to a cohort study, approximately 1/3 of PACA patients are unable to ambulate independently or maintain independent living 6 months after symptom onset.^[4]^ Early intervention cannot only stabilize symptoms but also rescue dysfunctional Purkinje cells.^[5]^

In this case, the patient exhibited progressive cerebellar atrophy and continued to require assistance with ambulation despite receiving sustained immunomodulatory therapy over a 17-month period. Notably, due to limited clinical recognition of autoimmune cerebellar ataxia, complementary and alternative treatment options were not considered until more than a year after receiving conventional therapy. Remarkably, following the initiation of acupuncture, the patient demonstrated significant improvement in ataxic symptoms. Even 1 year after discontinuation of acupuncture, she remained ambulatory without assistance and showed notable improvements in speech fluency.

Acupuncture, as a safe and cost-effective complementary therapy with minimal adverse effects, has shown promising efficacy in the treatment of cerebellar atrophy and ataxia.^[6]^ Based on Traditional Chinese Medicine Theory, the patient’s symptoms of dizziness, unsteady gait, bradykinesia, and slowed speech were considered manifestations of gallbladder Qi deficiency with functional disturbance. Wangu (GB12) and Fengchi (GB20) were selected to soothe the liver and regulate gallbladder Qi, thereby relieving vertigo and promoting cerebral circulation, and stimulation at these acupoints has been shown to suppress inflammatory factor expression, offering neuroprotective effects.^[7–9]^ Baihui (GV20) and Sishencong (EX-HN1) were used to elevate clear Yang, calm the mind, and enhance central nervous system function.^[10]^ Yanglingquan (GB34), Taichong (LR3), and Qiuxu (GB40) were employed to harmonize gallbladder meridian Qi and blood, facilitating limb coordination and balance.^[10,11]^ Mechanistically, stimulation of specific acupoints (e.g., Baihui [GV20], Shenting [HT7]) has been reported to reduce Purkinje-cell hyperexcitability and to promote Purkinje-cell survival, thereby preserving cerebellar structure and function after ischemic or inflammatory injury. This provides a plausible neuroimmune mechanism underlying the clinical improvements in motor function, speech, and mood observed in this case.^[12,13]^ This case highlights the value of incorporating complementary therapies, such as acupuncture, in treating autoimmune neurological conditions.^[14]^

At the 2-year follow-up the patient could walk independently (albeit slowly), which boosted her confidence and helped her engage more with others – leading to gradual improvements in speech fluency and clarity. She also reported less low mood, greater social participation, and a more positive outlook that meaningfully improved quality of life beyond what clinical scales show. Mechanistically, acupuncture may modulate central monoamine levels and activate neuro-signaling pathways, which can reduce depressive symptoms, promote social interaction, and potentially support recovery of speech function.^[15]^

However, this study has several limitations. Most notably, the patient continued immunosuppressive therapy (mycophenolate mofetil and prednisone) throughout the observation period, so a confounding effect of concurrent medical treatment cannot be excluded; therefore, causal attribution of the observed improvement to acupuncture alone is not possible – the symptomatic improvement most likely reflects the combined effects of acupuncture and ongoing immunotherapy. Most notably the lack of a control group, which limits the ability to draw causal inferences regarding treatment efficacy. Future research should employ controlled designs (for example, a 3-arm randomized trial comparing standard immunotherapy alone, immunotherapy plus early acupuncture, and immunotherapy with delayed/wait-list acupuncture), with adequate blinding, objective outcome measures, and prespecified timing of assessments to isolate the specific contribution of acupuncture and determine the optimal timing of its initiation (early vs delayed).

## 4. Conclusions

We report a case of PACA with notable improvement following combined immunomodulatory and acupuncture treatment. This case underscores the highlights the potential utility of acupuncture as a complementary approach in the management of PACA. Further research is warranted to validate the role of acupuncture in immune-mediated ataxias and explore underlying mechanisms.

## Acknowledgments

We sincerely thank the patient for their cooperation and willingness to share clinical information for this case report.

## Author contributions

**Conceptualization:** Linna Wu, Haolong Guo.

**Writing – original draft:** Zhijie Xu, Fan Xu.

**Writing – review & editing:** Guiping Li, Shu Wang.
